# Admitted to the U. S. Navy

**Published:** 1869-04

**Authors:** 


Admitted to the U. S. Navy.—We are happy to announce the admission of Dr. Dwight Dickinson of Buffalo, to the U. S. Navy, as Assistant Surgeon. For the last two years Dr. Dickinson has been acting House Physician and Surgeon to the Buffalo General Hospital, and graduated in medicine from the University of Buffalo at its last commencement. The Naval Board of Examiners require the highest standard of attainments, and so rigid are the tests, that only about eight out of one hundred who apply, are able to obtain its approbation. Dr. Dickinson had already distinguished himself in all the examinations of the college, and was regarded both by his class mates and the members of the faculty as first in his class. All who have known him in his position in the General Hospital, and all who have been associated with him in the medical college, will take much pleasure in his success.

